# Bacterial vaginosis is significantly associated with a higher risk of high-risk human papillomavirus persistence: a cohort study in Nairobi, Kenya

**DOI:** 10.3389/fgwh.2026.1825375

**Published:** 2026-07-02

**Authors:** Raymond Chibvongodze, Mutinda Cleophas Kyama, Lucy Muchiri

**Affiliations:** 1 Department of Human Pathology, Faculty of Health Sciences, University of Nairobi, Nairobi, Kenya; 2 Department of Medical Laboratory Sciences, Jomo Kenyatta University of Agriculture and Technology, Nairobi, Kenya

**Keywords:** Africa, bacterial vaginosis, cervical cancer, cohort study, human papillomavirus, Kenya, risk ratio

## Abstract

**Background:**

Reasons why some human papillomavirus infections persist while others spontaneously resolve remain elusive. There is speculation that bacterial vaginosis increases the likelihood of high-risk human papillomavirus persistence. However, the magnitude of the risk it imposes on human papillomavirus infected women, which influences persistence, remains unclear. The objective of this study was to determine whether bacterial vaginosis is associated with a higher risk of persistent high-risk human papillomavirus infection among women.

**Methods:**

A total of 498 women were recruited at baseline, and of these, 97 women with a current human papillomavirus infection were enrolled into two cohorts, classified by baseline bacterial vaginosis status. The women were retested for both infections after 24 months.

**Results:**

The persistence of high-risk human papillomavirus was more common in women with baseline bacterial vaginosis (*p* = 0.01), and type HPV-52 was the most persistent genotype. The risk of persistence was 1.38 times greater among women with baseline bacterial vaginosis than among those without. Multivariate logistic regression analysis revealed that BV resolution influenced whether HPV persisted or was cleared in this study (aOR = 5.40, 95%CI = 2.24–13.07; *p* = 0.002).

**Conclusion:**

These findings indicate greater biological susceptibility to high-risk human papillomavirus persistence among women with bacterial vaginosis. This result highlights the significance of effectively treating BV when it occurs, as doing so may assist in reducing the risk of hr-HPV persistence.

## 1 Introduction

Infection by persistent high-risk human papillomavirus (hr-HPV) is regarded as a prerequisite for the development of cervical cancer and its precancerous lesions (1). In 2020, the prevalence of human papillomavirus (HPV) in Kenya was reported to be 23.6% (2). However, most hr-HPV infections are transient and resolve spontaneously within 24 months; only a minority persist (3). HPV persistence is defined as the detection of the same HPV genotype(s) on two consecutive consultation visits (4). However, why some hr-HPV infections persist while others spontaneously resolve remains elusive (5); therefore, co-factors for HPV persistence have not been fully elucidated. According to Zhao et al.*,* the majority of transient HPV infections resolve within 24 months, and they recommended a 24-month interval to be used as the indicator to define a persistent HPV infection (6). Therefore, in this study, the detection of the same hr-HPV genotype(s) after 24 months of follow-up was regarded as hr-HPV persistence.

There is speculation that bacterial vaginosis (BV), a common polymicrobial vaginal disorder characterized by depletion of *Lactobacillus* species and subsequent replacement by an overgrowth of anaerobic bacteria, plays a role in increasing the risk of HPV persistence (7). However, this hypothesis has not been proven in a properly designed cohort study, and the magnitude of the risk of BV-associated HPV persistence has remained elusive. A few previous cohort studies that attempted to investigate this concept yielded controversial findings for several reasons, including poor external validity (a study that began with pregnant participants) (8) and poor objectivity (due to the utility of Pap smears for detecting BV status) (5).

BV is believed to contribute to hr-HPV persistence through several biological mechanisms. One proposed pathway involves the production of elevated levels of nitrosamines in the vaginal environment as a result of BV (7). These nitrosamines are thought to induce changes in the local immune response, specifically by altering the cytokine profile. Increased concentrations of interleukins namely IL-1, IL-6, IL-8, and IL-10 are observed under these conditions (7). The shift in the cytokine profile associated with BV may weaken the immune system's capacity to effectively clear and eliminate newly acquired HPV infections (7). As a consequence, this impairment in immune response can lead to the persistence of HPV within the host. In addition, BV is believed to induce a proinflammatory environment that facilitates the expression of early HPV genes (*E6* and *E7*), which spearhead viral integration into the host genome, genomic instability, and telomerase activation, all of which are necessary for cervical carcinogenesis (9).

The possibility of BV being a co-factor for hr-HPV persistence is a major public health concern in Africa as the region is overburdened by both infections and has the highest rates of cervical cancer in the world. In the present cohort study, we aimed to investigate whether BV is associated with a higher risk for hr-HPV persistence. Since the study was done in a reproductive health clinics, Pap smear cytological findings and their correlation with hr-HPV were discussed as a secondary objective in this study.

## 2 Materials and methods

### 2.1 Study design

This was a cohort study with the broad objective of investigating whether BV is associated with an increased risk of hr-HPV persistence. At baseline, 498 women were enrolled and cotested for BV and hr-HPV infections. Based on baseline BV status, two cohorts were formed. The two cohorts were the BV-positive cohort (BV positive/hr-HPV positive results at baseline assessment) and the BV-negative cohort (BV negative/hr-HPV positive results at baseline). Women in both cohorts were followed up for a minimum of 24 months and retested for BV and hr-HPV. Each cohort initially included 49 women, unfortunately one woman in the BV positive cohort declined follow up testing, therefore, 97 women (48 in the BV-positive and 49 in the BV-negative cohorts) were investigated further in the follow up phase. This study tested women for the presence or absence of BV/hr-HPV at only two time points (baseline and follow up). The cohort study began in July 2022 and concluded in November 2025.

### 2.2 Study setting

The study was conducted at Kenyatta National Hospital (KNH) Reproductive Health clinics, Kenya AIDS Vaccine Initiative-Institute of Clinical Research (KAVI-ICR), KNH cytology lab, Hercules Medical Laboratory, and Kenya Medical Research Institute (KEMRI).

### 2.3 Study population and eligibility for enrollment

The follow-up phase of the study involved retesting women with baseline results that qualified them for either the BV-positive cohort (BV-positive/hr-HPV-positive) or the BV-negative cohort (BV-negative/hr-HPV-positive). All women in the follow up phase were older than 30 years. Women with high-grade squamous intraepithelial lesions (HSILs) at baseline were excluded from the follow-up phase.

At baseline, this study recruited 498 women who had voluntarily reported to reproductive health clinics for Pap-based cervical cancer screening. After baseline testing, results were given to the clinics and appropriate clinical decisions were made. The authors were not involved in the clinical decisions or management of the patients. The clinic continued to follow up the women at intervals stipulated by their established clinical guidelines. The women 401 who did not qualify for the follow up phase continued to receive follow up or monitoring services in the clinic and were not in any way disadvantaged. This study was an observational cohort study that only assessed for the presence of both BV and hr-HPV at baseline and at 24 months without interfering in the routine management schedules of the patients.

### 2.4 Data collection and sample collection

Data were gathered through face-to-face interviews using a structured questionnaire with closed-ended questions. The following data were gathered: age (years), follow-up period (months from baseline assessment), Human Immunodeficiency Virus (HIV) status, number of sexual partners over the past two years, douching practice and date of the last menstrual period (LMP). All women signed consent forms and samples were collected as described in (10).

### 2.5 Laboratory processes

#### 2.51. PCR detection of hr-HPV

hr-HPV DNA testing was performed via the commercial Sacace HPV Genotypes 14 Real-TM Quant test kit (CE-IVD) (Sacace Biotechnologies, Como, Italy), which detects fourteen high-risk genotypes: 16, 18, 31, 33, 35, 39, 45, 51, 52, 56, 58, 59, 66, and 68. The procedures for HPV DNA extraction, amplification and detection were described in (10).

#### 2.5.2 PCR detection of BV

BV detection was carried out via the commercial Vircell Vaginal Panel Real-time PCR Kit (CE-IVD) (Vircell Molecular, Granada, Spain), which detects BV on the basis of the relative quantities of three markers: *Lactobacillus* species*, Gardnerella Vaginalis, and Atobium Vaginae.* The procedures for bacterial DNA extraction, amplification, detection, and interpretation were described in (10).

#### 2.5.3 Pap smear processing and interpretation

Pap smears were stained with Papanicolaou stain and evaluated by two independent primary individuals, a cytologist and a pathologist. Discrepant findings were referred to a third person, a pathologist with extensive experience in cytopathology. The 2014 Bethesda System for reporting cervical cytology was used to report the cytology findings (11). Patients with abnormalities were referred to a gynecologist within the same clinic for further management.

### 2.6 Statistical analysis

#### 2.6.1 Sample size determination

Sample size was determined via the Fleiss JL formula for cohort studies. Data from a study by Dahoud et al. were used to calculate the sample size (12). In Dahoud's study, the proportion of BV-positive women among HPV-positive women (P1) was 20.3%, and the proportion of BV-negative women among HPV-positive patients (P2) was 79.6%. The study power was set at 90%, the level of significance at 10%, and a 10% attrition rate was factored in. A minimum of 14 patients per cohort (BV-positive and BV-negative) was required. However, in this study, 48 women in the BV-positive cohort and 49 women in the BV-negative cohort were followed up.

A larger sample size in both cohorts was used to enhance statistical power, give better representation of heterogeneity, and increase precision. The larger sample size was also used to account for potential dropouts, non-responses, or missing data to ensure a final usable sample size capable of generating conclusions that are more convincing, reliable, and representative of the population.

#### 2.6.2 Study variables

Age and follow-up period were captured as continuous variables. The LMP was captured as the actual date in the format (dd/mm/yyyy). Women were classified as younger women (≤41 years old) (median age) or older women (>41 years old). The number of sexual partners was dichotomized into ≤1 or ≥2. HIV status was captured as negative, positive or unknown (having not tested over the past 2 years). Douching practice was dichotomized into “yes” and “no”.

All women enrolled in the follow-up phase were hr-HPV positive at baseline. A woman was deemed to have a persistent hr-HPV infection if any of the hr-HPV genotype(s) previously detected at baseline were detected again at follow-up testing, regardless of whether the woman had other new hr-HPV genotypes at follow-up. Conversely, a woman was considered to have cleared hr-HPV if the hr-HPV genotype(s) detected at baseline were not detected again at follow-up testing, regardless of whether the woman had other new hr-HPV genotypes. Table 1 below illustrates workable examples of this.

**Table 1:** Interpretation of persistence or clearance status (examples).

Baseline hr-HPV genotypes	Follow up genotypes	Interpretation
HPV **33**, 56, 68	HPV 66, **33**, 45	Persistent hr-HPV infection
HPV 16, 31,35	HPV 18, 52	Cleared hr-HPV infection

Bold hr-HPV genotypes are those detected at both baseline and follow-up testing.

#### 2.6.3 Data processing and statistical tests

The data gathered via a paper-based questionnaire were coded before being entered into Microsoft Excel, then exported to SPSS version 29 for cleaning and analysis. Categorical variables are presented as frequencies and percentages. Continuous variables are reported as means ± standard deviations, medians, and ranges.

The risk ratio (RR) was used to quantify the risk in the BV-positive cohort relative to the BV-negative cohort. Two-sided independent *t* tests were used to compare the hr-HPV persistence rates among younger women (≤41 years) and older women (>41 years), to compare the hr-HPV persistence rates among women in the BV-positive cohort group and the BV-negative cohort after 24 months of follow-up, and to compare the hr-HPV persistence rates among women who had or did not have BV at the follow-up visit. Multivariate logistic regression analysis was conducted to assess the adjusted strength of the association between BV and hr-HPV persistence, and to control for potential confounders simultaneously. The primary variables for adjustment were age, vaginal douching practice, number of sexual partners, and HIV status because they were independently associated with either BV or hr-HPV at baseline assessment (10). The logistic regression results are presented as adjusted odds ratios (aORs) and 95% confidence intervals (CIs). In all analyses, a *p*-value <0.05 was considered statistically significant.

### 2.7 Ethical statement

This study was approved by the Kenyatta National Hospital–University of Nairobi Ethics and Research Committee (KNH-UoN ERC), reference number: (P107/02/2019). Written informed consent for participation and the publication of anonymized patient data was obtained from all study participants.

## 3 Results

**Figure 1:**
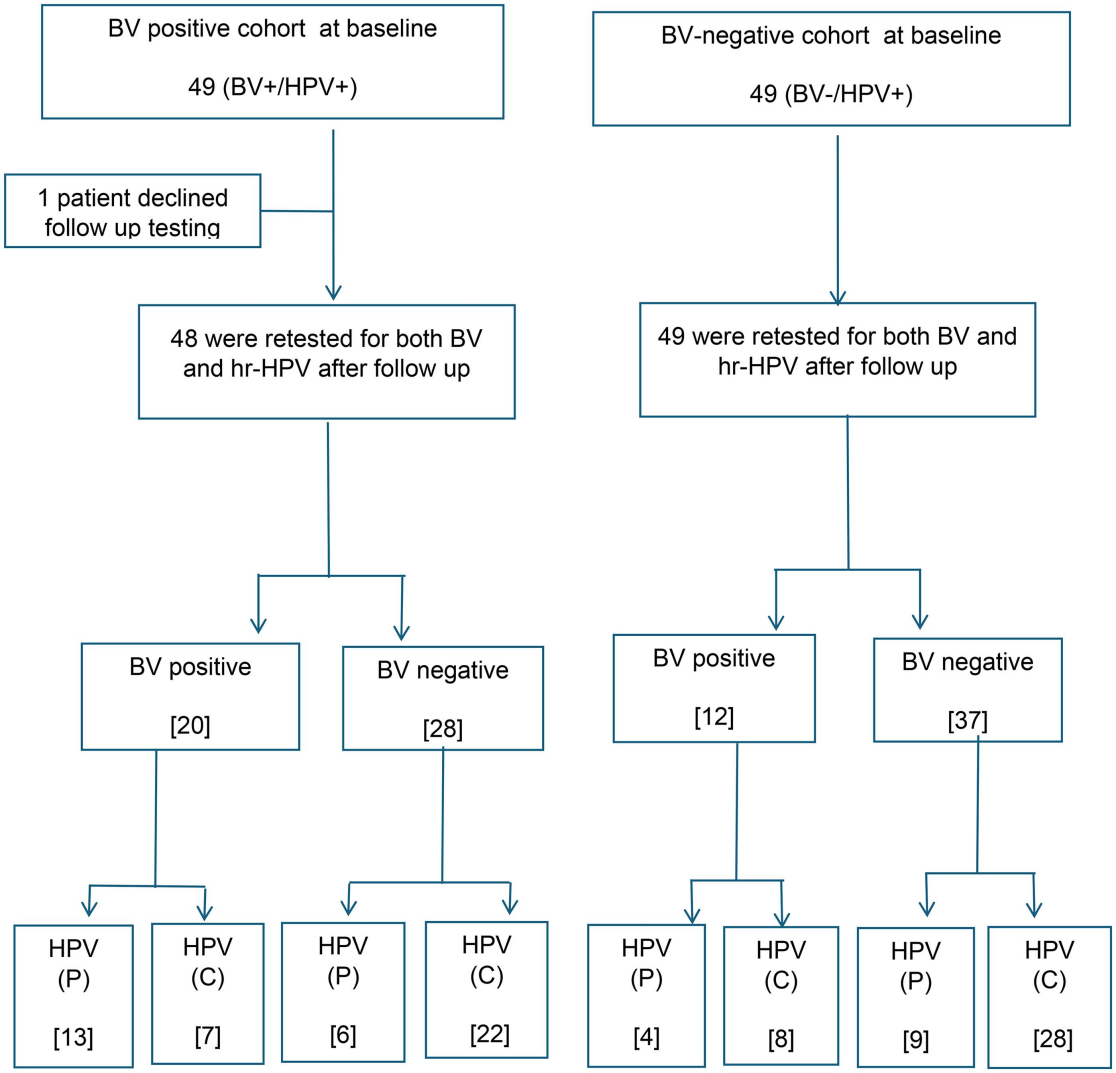
Flow chart of the study protocol. HPV (P), Patients with a persistent hr-HPV infection; HPV (C), Patients who cleared an hr-HPV infection.

At the follow-up phase of the cohort study, a total of 97 patients classified by their baseline status, namely, the BV-positive cohort (48 women) and the BV-negative cohort (49 women), were followed up and retested for both BV and hr-HPV after a minimum of 24 months. The number of women in both cohorts were 49 initially. Unfortunately, one woman in the BV-positive cohort declined follow-up testing. The flowchart for the follow-up phase of this cohort study is shown in Figure 1.

### 3.1 Age and follow-up period of the study participants

The mean (SD) age of the 97 participants in this study was 43.1 (8.9) years, with a median (range) of 41 (32–68) years. The majority of women (58.8%, 57/97) were ≤41 years old (median age), and 41.2% (40/97) were older than 41 years. Among the 57 women ≤41 years, 23 (40%) had persistent hr-HPV, and among the 40 women >41 years, 10 (25%) had persistent hr-HPV after follow-up testing. Therefore, hr-HPV persisted more often among younger women than among older women [40% (23/57) vs. 25% (10/40), respectively; *p* = 0.02]. The follow up ranged from 24 to 28 months with an average of 24.5 months. The follow-up period for women in the BV-positive cohort was longer than that for women in the BV-negative cohort (25.1 vs. 24.3 months, *p* = 0.008).

### 3.2 Follow-up BV/hr-HPV cotesting results (after a minimum of 24 months)

As shown in Figure 1 below, 32 of the 97 cotested women (33.0%) had persistent hr-HPV infections (the same hr-HPV genotype was detected at both baseline and follow-up testing). Among the 48 women who had BV at baseline (BV-positive cohort), 58.3% (28/48) had resolved the BV, and 41.7% (20/28) had unresolved BV infections following follow-up testing. Among the 49 women who were BV-free at baseline (BV-negative cohort), new/incident BV infections were detected in 24.4% (12/49) of the women. Therefore, a total of 32 out of 97 women were reported to have BV at follow-up, and of these 32 women, 17 (53.1%) also had hr-HPV persistence. Among the remaining 65 women who were BV-free at follow-up, only 15 (21.3%) had persistent hr-HPV. Therefore, more hr-HPV cases persisted when BV was present at follow-up [53.1% (17/32) vs. 21.3% (15/65), *p* *<* 0.001]. These results are summarized in Table 2 below.

**Table 2:** Comparison of hr-HPV persistence in women with or without BV at follow-up (after 24 months).

BV at follow up testing	*N*	hr-HPV Persistence rate	*t*	df	*p*
BV-positive	32	53.1% (17/32)	3.071	95	<0.001
BV-negative	65	21.3% (15/65)			

After a minimum of 24 months of follow-up, a *t-*test revealed that hr-HPV persisted more often in the BV-positive cohort (BV-positive/hr-HPV-positive at baseline assessment) than in the BV-negative cohort group (BV-negative/hr-HPV-positive at baseline assessment) (*p* = 0.01). These results are summarized in Table 3 below.

**Table 3:** Results of *t* tests for comparisons of hr-HPV persistence between the BV-positive and BV-negative cohorts after 24 months.

Cohort group	*N*	hr-HPV Persistence rate	*t*	df	*p*
BV-positive	48	39.6% (19/48)	1.366	95	0.01
BV-negative	49	26.5% (13/49)			

The forty-eight women who had BV/hr-HPV coinfection at baseline (BV-positive cohort participants) were evaluated for BV resolution and hr-HPV persistence or clearance. 46% (22/48) of the women had cleared hr-HPV and resolved baseline BV within 2 years. However, 27.1% (13/48) still had both persistent hr-HPV infection and unresolved BV infection. Multivariate logistic regression analysis revealed that BV resolution influenced whether HPV persisted or was cleared (aOR=5.40, 95% CI = 2.24–13.07; *p* = 0.002). The cross-tabulation of hr-HPV persistence/clearance and BV resolution is shown in Table 4.

**Table 4:** Cross-tabulation of BV status and outcome of interest (hr-HPV persistence).

Cohort group	hr-HPV persistence	hr-HPV clearance	RR
BV positive	19	29	1.38
BV negative	13	36	

### 3.3 Risk ratio for hr-HPV persistence between the BV-positive cohort and the BV-negative cohort

Table 5 below shows BV status at the baseline assessment and the outcome of interest (HPV persistence) at the end of the study. The rate of hr-HPV persistence among the BV-positive cohort was 39.6% (19/48), or 40 per 100 individuals, over 2 years. The rate of hr-HPV persistence in the BV-negative cohort was 28.6% (13/49), or 29 per 100 individuals over 2 years. The risk ratio (RR) between the BV-positive and BV-negative cohorts was 1.38 (39.6/28.6). Therefore, the risk of hr-HPV persistence was 1.38 times greater in the BV-positive cohort than in the BV-negative cohort over 2 years.

**Table 5:** Association between BV resolution and hr-HPV persistence/clearance.

BV status	HPV clearance	HPV persistence	OR	95% CI	*p*
Resolved BV	22	6	6.81	1.88–24.69	0.002
Unresolved BV	7	13			

### 3.4 Persistence pattern of specific hr-HPV genotypes in this study

At baseline, a total of 149 hr-HPV genotypes were detected in women who met the study's follow-up phase criteria. Among these 149 cases, only 38 were detected in the same women at follow-up, yielding an overall hr-HPV persistence rate of 25.5% in this study. At the specific hr-HPV genotype level, HPV-52 was the most persistent hr-HPV genotype (57.1%), and HPV-18 and HPV-59 were the least persistent genotypes (0%). The persistence rates of all the other genotypes are summarized in Table 6 below.

**Table 6:** hr-HPV-specific persistence rates'.

hr-HPV genotype	Baseline frequency (in 97 patients)	Follow up frequency (in 97 patients)	Persistence rate (%)
16	4	1	25.0%
18	1	0	0.0%
31	17	7	41.2%
33	12	1	8.3%
35	13	3	23.1%
39	17	4	23.5%
45	9	5	5.6%
51	11	3	27.3%
52	7	4	57.1%
56	22	3	13.6%
58	12	3	25.0%
59	4	0	0.0%
66	12	3	25.0%
68	8	1	12.5%
Overall	149	38	25.5%

### 3.5 Pap smear results

Most of the women (92.8%, *n* = 90/97) had negative for intraepithelial lesion or malignancy (NILM) cytology results. No HSIL lesion or higher was identified at follow-up testing. The remaining results are displayed in Table 7 below. Atrophy was the most reported non-neoplastic finding in this study (10.3%, 10/97). Twenty-two (22.7%) of the Pap smears analyzed in this study showed features suggestive of BV; of these, only 10 were confirmed by PCR, yielding a positive predictive value (PPV) of 45.5% for detecting BV by Pap smear. No *Trichomonas vaginalis* was identified in the follow-up phase of the study. Of the 5 women with baseline atypical squamous cells of undetermined significance (ASC-US) or low grade squamous intraepithelial lesion (LSIL) results who were eligible for enrollment in the follow-up phase, 3 regressed to negative for intraepithelial lesion or malignancy (NILM). The other two women had ASC-US and LSIL results each (Table 7) and were recommended for further management at the same clinic.

**Table 7:** Pap smear findings.

Interpretation	*N*	Frequency (%)
Unsatisfactory	1	1
NILM	90	92.8
ASC-US	4	4.1
LSIL	2	2.1
Non-Neoplastic Findings		
Shift in flora suggestive of BV	22	22.7
Fungal organisms consistent with *Candida* spp.	6	6.2
Atrophy	10	10.3
Reactive cellular changes associated with Inflammation and typical repair	8	8.2

NILM, Negative for intraepithelial lesion or malignancy; ASC-US, Atypical squamous cells of undetermined significance; LSIL, Low-grade squamous intraepithelial lesion.

### 3.6 Pap smear/pooled hr-HPV result combinations at follow up visit (after 24 months)

The majority of the women (64.9%, 63/97) had NILM/hr-HPV negative result combinations. Eighty three percent (5/6) of women with ≥ASCUS cytology results were positive for hr-HPV. The remaining result combinations are summarized in Table 8 below. There was a statistically significant association between cytology and pooled hr-HPV status (*p* = 0.044) (Table 8).

**Table 8:** Pap smear cytology/pooled hr-HPV result combinations at follow up testing.

hr-HPV		Pap smear results				*X^2^*	df	*p*
		Unsatisfactory	NILM	ASC-US	LSIL	8.11	3	0.04
hr-HPV	Neg	1	63	1	0			
	Pos	0	27	3	2			

## 4 Discussion

The present study was a cohort study aimed at investigating whether BV is associated with a higher risk of hr-HPV persistence. This study revealed that hr-HPV persisted more often in the BV-positive cohort than in the BV-negative cohort and that women in the BV-positive cohort had a greater risk (1.38 times) of having hr-HPV persistence than women in the BV-negative cohort did after 24 months of follow-up. It was also demonstrated in this study that BV resolution influenced whether HPV persisted or cleared. Additionally, this study revealed that more hr-HPV cases persisted when BV was present at the follow-up visit.

For several decades, persistent hr-HPV infection has been known to be a proven cause of cervical intraepithelial neoplasia (CIN) and cervical cancer (1), but why some hr-HPV infections persist, and others are self-resolving, remains elusive (5). The findings from this cohort study confirmed that BV is among the cofactors associated with the persistence of an hr-HPV infection. These findings are consistent with those of Gillet et al. and King et al., who reported that BV is a potential cofactor in HPV persistence (13, 14). However, unlike these two studies, which hypothesized this concept, the present study aimed to prove that the hypothesis was correct.

The mechanisms underlying BV-induced HPV persistence remain poorly understood. BV is believed to contribute to the persistence of hr-HPV infections through several biological mechanisms. One proposed pathway involves the production of elevated levels of nitrosamines in the vaginal environment as a result of BV (7). These nitrosamines are thought to induce changes in the local immune response, specifically by altering the cytokine profile. Increased concentrations of interleukins—namely IL-1, IL-6, IL-8, and IL-10 are observed under these conditions (7). The shift in the cytokine profile associated with BV may weaken the immune system's capacity to effectively clear and eliminate newly acquired HPV infections (7). As a consequence, this impairment in immune response can lead to the persistence of HPV within the host, thereby increasing the risk of ongoing infection. In addition, BV is believed to induce a proinflammatory environment that facilitates the expression of early HPV genes (*E6* and *E7*), which spearhead viral integration into the host genome, genomic instability, and telomerase activation, all of which are necessary for cervical carcinogenesis (9).

However, these are still hypothetical mechanisms that need to be proven in well-designed studies. Brotman et al. reported an interesting perspective to the BV-HPV relationship. According to Brotman *et al*, the association between BV and HPV persistence could be due to both infections sharing a common pathophysiology (reduced levels of hydrogen peroxide-producing *Lactobacilli*, which would normally inhibit both bacterial and viral infections) or infections that merely share common risk factors such as age, sexual practices, number of sexual partners, smoking, oral contraceptive use, parity, etc. (15). However, at baseline assessment, the authors investigated the risk factors for both BV and HPV, and there were no common risk factors for either BV or HPV in this study population (10).

The association of BV with a higher risk for hr-HPV persistence in this study has profound clinical significance. These findings underscore the importance of thorough treatment of incident BV infections to promote hr-HPV clearance. This finding was supported by Guo *et al*., who advocated simple, economical ways to promote hr-HPV clearance, such as the application of vaginal *Lactobacillus* probiotics (5). Additionally, McDermott reported that a probiotic (Papilocare) is already on the market in Europe and has been shown in a randomized clinical trial to enhance hr-HPV clearance (16).

Another noteworthy finding was a higher hr-HPV persistence rate in younger women (≤41 years old) than in older women (>41 years old). This contradicts the findings of Zhao et al.*,* who reported that older women were more susceptible to persistent hr-HPV infections than younger women were (6). Zhao et al. attributed this to a decrease in immune system competency with aging, which could aid hr-HPV in evading detection and elimination by host cell-mediated immunity (6).

This study had some major strengths. The potential link between BV and HPV persistence remains under-explored, and a few cohort studies that attempted to investigate it have yielded controversial findings due to subjective methods used to detect BV (5, 8). This shortfall was overcome in this study by the utility of PCR to detect BV, which improved objectivity and minimized interobserver variability. Second, this study was the first cohort study to investigate and document the magnitude of the risk that BV imposes on HPV-infected patients, influencing hr-HPV persistence relative to patients with hr-HPV infection but without BV. The authors, however, wish to to disclaim that there could be an under or overestimation of the reported magnitude due to the ever-dynamic nature of vaginal microbiota. Thirdly, this study employed a follow up period of 24 months. This interval was determined in a study by Zhao et al. (6) to be the best follow up period to determine persistent infections. This makes the findings from this study both credible and superior to other studies that used shorter follow up periods. However, in this study, a few women were followed up a few months after the 24th month ideal interval due to the women having tight schedules.

This study has a few major limitations. First, the cohort study design was complex because we relied on baseline BV exposure status to construct the BV-positive and BV-negative cohorts. However, the vaginal microbial ecosystem is ever dynamic, and women classified as BV-positive may have cleared BV shortly after enrolment or the reverse. This dynamism may have introduced a risk for exposure misclassification. Additionally, there was no guarantee that these statuses will be maintained over 2 years. This limitation was also acknowledged by Menon et al.*,* who highlighted the ever-dynamic vaginal microbiota as the major impediment to establishing inferences among BV, HPV infection, HPV persistence, and cervical cancer (17). Similarly, the authors used the terms “resolved” and “unresolved” BV in this study. However, given the dynamic nature of the vaginal microbiota over time, this should be considered only an assumption based on BV results at the two patient contact points. Despite this limitation, the authors are optimistic that this study yielded credible conclusions that will contribute significantly to the existing literature. The other limitation concerned the authors' role in this study. The investigators were mere observers and had no control over clinical decisions taken after baseline testing. They were therefore, unaware of whether treatment was administered or not. This deviated from the established principles of a randomized clinical trial (RCT). To further investigate inferences between BV and HPV persistence, the authors therefore, recommend a similar study involving a BV treatment intervention and more frequent follow ups.

At baseline assessment, it was challenging to know which infection was acquired first, and temporality was uncertain. Therefore, reverse causality between BV and hr-HPV persistence cannot be excluded. Reverse causality was supported by Lebeau et al., who postulated that HPV infection alters the vaginal microbiome by inhibiting the expression of host mucosal innate peptides, which are amino acid sources for *Lactobacilli*, resulting in the depletion of lactic acid-producing *Lactobacilli* (18). Therefore, a cohort study designed to investigate whether HPV infection is a co-factor in the development of BV is recommended to rule out reverse causality.

## 5 Conclusions

The study found that the risk of hr-HPV persistence was 1.38 times higher among women in the BV-positive cohort compared to those in the BV-negative cohort group, when observed over a period of two years. These findings illustrate a greater biological susceptibility to hr-HPV persistence in women with BV. This result highlights the significance of effectively treating BV when it occurs, as doing so may help reduce the risk of hr-HPV persistence.

## Data Availability

The raw datasets used and/or analyzed during the current study are available from the corresponding author on reasonable request.
